# Canonical and Non-canonical Genomic Imprinting in Rodents

**DOI:** 10.3389/fcell.2021.713878

**Published:** 2021-08-05

**Authors:** Hisato Kobayashi

**Affiliations:** Department of Embryology, Nara Medical University, Kashihara, Japan

**Keywords:** genomic imprinting, DNA methylation, non-canonical imprinting, histone modification, rodent, germline differentially methylated region, mouse genome, epigenetics

## Abstract

Genomic imprinting is an epigenetic phenomenon that results in unequal expression of homologous maternal and paternal alleles. This process is initiated in the germline, and the parental epigenetic memories can be maintained following fertilization and induce further allele-specific transcription and chromatin modifications of single or multiple neighboring genes, known as imprinted genes. To date, more than 260 imprinted genes have been identified in the mouse genome, most of which are controlled by imprinted germline differentially methylated regions (gDMRs) that exhibit parent-of-origin specific DNA methylation, which is considered primary imprint. Recent studies provide evidence that a subset of gDMR-less, placenta-specific imprinted genes is controlled by maternal-derived histone modifications. To further understand DNA methylation-dependent (canonical) and -independent (non-canonical) imprints, this review summarizes the loci under the control of each type of imprinting in the mouse and compares them with the respective homologs in other rodents. Understanding epigenetic systems that differ among loci or species may provide new models for exploring genetic regulation and evolutionary divergence.

## Rodents: Symbolic Models in Biomedical and Genetic Research

Rodents such as mice and rats are commonly used as representative laboratory animals. The genomes of these organisms have been progressed along with the human genome project; thus, the C57BL/6 mouse (*Mus musculus*) and Brown Norway rat (*Rattus norvegicus*) become the second and third mammals to have their genomes sequenced in 2002 and 2004, respectively (Waterston et al., 2002; Gibbs et al., 2004). Their genomes of approximately three billion base pairs each contains roughly the same number of genes as the human genome. Furthermore, almost all human genes associated with diseases have counterparts in the rodent genome, which appear highly conserved throughout mammalian evolution. Thus, these experimental rodents generally deepen our understanding of mammalian genetic and (epi-)genomic regulatory systems.

Mammals are diploid organisms arising from the fusion of two parental gametes, with each donating one set of autosomal chromosomes (19 autosomes in mice, 20 in rats, and 22 in humans) plus one set of sex chromosome (X or Y) to the offspring. According to Mendel’s law, diploid cells contain parental copies of each autosomal gene, which are predicted to show the same transcription state. However, “genomic imprinting” is a form of non-Mendelian inheritance that results in parent-of-origin allele-specific gene expression of autosomal loci or of loci on the diploid X chromosome (only in extra-embryonic tissues in females). Polymorphism information between strains or individuals helps distinguish between paternal and maternal alleles. It was only natural that the phenomenon of genomic imprinting was discovered and well-investigated in mice, where nuclear transfer and genetic engineering technologies have always been developed and where numerous strains (polymorphisms between strains can serve as genetic markers of parent-of-origin in allele-specific analysis) have been established and maintained.

## Discovery of Genomic Imprinting in Mammals

In 1984, two laboratories published landmark papers that reported a new phenomenon in mammalian genome biology (Barton et al., 1984; McGrath and Solter, 1984). Both studies independently performed pronuclear transfer experiments from fertilized mouse eggs to produce androgenetic and gynogenetic embryos containing only sperm-derived or oocyte-derived chromosome sets. These “uniparental” embryos could not survive to term but could develop to some extent with sex-specific developmental abnormalities. Androgenetic embryos preferentially develop extra-embryonic and placental structures at the expense of embryo development. Conversely, gynogenetic embryos (or parthenogenetic embryos by artificial activation of oocytes) have poor growth of placental lineages and developmental arrest, possibly due to extra-embryonic defects. These opposite phenotypes underlie the functional differences in developmental genes in paternal and maternal genomes. The mice that were bred to have uniparental disomies, in which either single or partial chromosomes are inherited from only one parent, for individual chromosomes also show aberrant phenotypes, such as overgrowth, growth retardation, or abnormal behavior (Cattanach, 1986).

Nevertheless, not all chromosomes produce abnormalities when present as disomies, depending on which chromosome or part is made uniparental; however, those commonly lead to striking phenotypic differences. These investigations revealed the requirement of both maternal and paternal genomes for normal development, which was tied to an intriguing biological phenomenon called genomic imprinting. Uniparental inheritance of the genome or chromosome occurs spontaneously in humans, resulting in early pregnancy losses, like androgenetic and parthenogenetic conceptuses (hydatidiform moles and benign ovarian teratomas), or moderate to severe developmental disabilities, known as imprinting diseases (Linder et al., 1975; Kajii and Ohama, 1977; Wake et al., 1978; Kalish et al., 2014). Subsequent evolutionary and genetic studies of imprinted loci have shown that this phenomenon is present only in placental mammals among vertebrates.

The surprising finding of these studies was that mammalian genes could function differentially depending on whether they originated from the mother or father. Before the study of uniparental disomies, a “maternal-effect” locus called *Tme* (*T-associated maternal effect*) was identified on the proximal mouse chromosome 17 overlapping deletions of maternal-effect lethal mutants, like *T*^*hp*^ or *T^*lub*2^* (Johnson, 1974; Winking and Silver, 1984). The region was later revealed to be the locus of *Igf2r*, expressed exclusively from the maternally inherited allele; therefore, its expression is dependent on the “parent-of-origin.” Simultaneously, the closely linked *H19* and *Igf2* genes, which are reciprocally imprinted, were identified in mouse chromosome 7; *H19* produces a long non-coding RNA (lncRNA) exclusively expressed from the maternal allele, and *Igf2* originates from the opposite allele. Interestingly, the opposite imprinting of *Igf2* and its scavenging receptor gene, *Igf2r*, demonstrates conflicting parental effects of growth promotion and growth restriction, which supports the classic “parent-offspring conflict theory” for the evolution of genomic imprinting (Trivers, 1974; Moore and Haig, 1991).

## Canonical Genomic Imprinting Is Mediated by Maternal or Paternal DNA Methylation

The discovery of the first endogenous imprinted genes in 1991 (Barlow et al., 1991; Bartolomei et al., 1991; DeChiara et al., 1991), which were differentially expressed from the maternal and paternal alleles, sparked initial efforts to elucidate the mechanisms of imprint establishment, maintenance, and erasure that together control the timing and placement of genomic imprinting. One prominent candidate of the non-Mendelian system is epigenetic regulation, in which DNA methylation (mainly occurs in CpG dinucleotides) is the most studied mechanism and has been shown to play a key role in mouse models of genomic imprinting and fetal reprogramming. A strong link between DNA methylation and imprinting regulation has been indicated by the cases of imprinted transgenic mouse lines. In a few of these mice the foreign transgene becomes methylated in a parent-specific manner in the gamete, inherited with parent-of-origin specific methylation into the diploid cells of embryo, and subsequently, the modification is erased and reestablished upon passage through the germ line (Chaillet et al., 1991).

**FIGURE 1:**
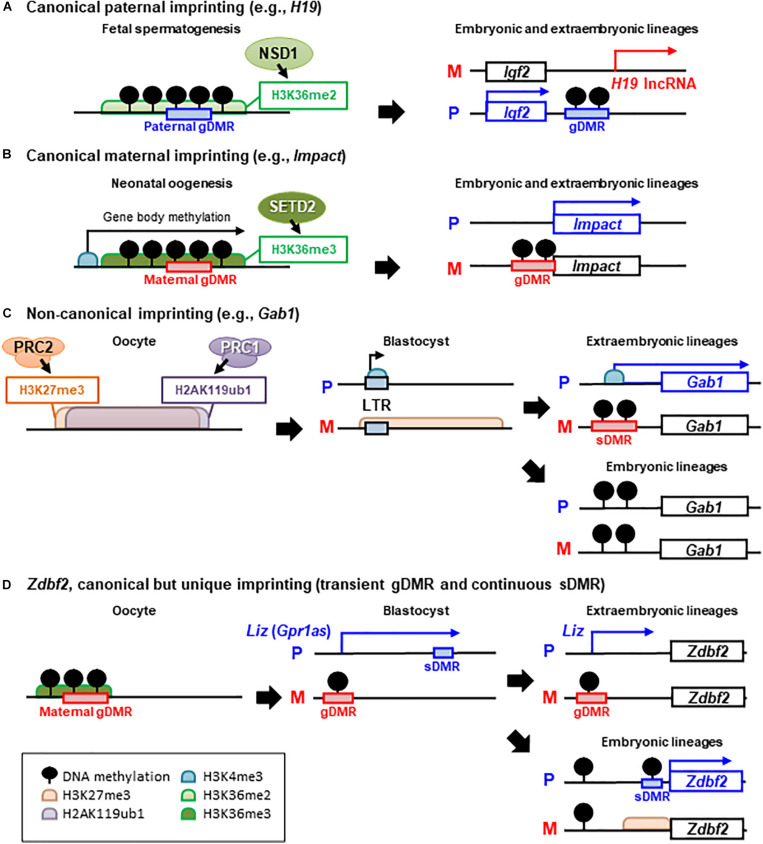
Epigenetic mechanisms of canonical and non-canonical imprinted gene regulation. There are several examples of different epigenetic inheritance patterns between paternal (P) and maternal (M) alleles, that control paternally expressed protein-coding genes. **(A)** Paternal and **(B)** maternal germline differentially methylated region (gDMR)-mediated canonical imprinting. H3K36 methyltransferases NSD1 and SETD2 have been shown to establish H3K36me2 or H3K36me3 in pro-spermatogonia or oocytes and to be required for *de novo* DNA methylation at paternal or maternal gDMRs, respectively. In general, canonical imprinting is stably maintained throughout somatic life and mediates monoallelic silencing of imprinted genes or non-coding RNAs. **(C)** Non-canonical imprinting, such as *Gab1* loci. H2AK119ub1 guides maternal inheritance and zygotic deposition of H3K27me3, and thus, maternally inherited H3K27me3 is maintained until the blastocyst (pre-implantation) stage. Then, maternal H3K27me3 silences the LTR retrotransposon-derived alternative promoter, which becomes actively transcribed on only the paternal allele. Although maternal H3K27me3 is lost after implantation, maternal allele-specific DNA methylation is established as an imprinted sDMR in extra-embryonic tissues, and thus, monoallelic paternal expression of non-canonically imprinted LTRs and nearby protein-coding genes can be maintained. In the post-implantation epiblast, these ERVs are silenced by DNA methylation in both alleles, resulting in a loss-of-imprinting in somatic lineages (not shown in the figure). **(D)** *Zdbf2* locus is a unique example of secondary imprinting. Transient paternal allele-specific expression of a long isoform transcript of *Zdbf2* (*Liz*, also called *Gpr1as*) originated from the maternal gDMR, occurs in the pre-implantation embryo. *Liz* continues to be paternally expressed by the persistence of the maternal gDMR in extra-embryonic tissues. In the embryonic tissues, maternal gDMR is biallelically methylated and loses its imprinted status and *Liz* transcription; However, *Zdbf2* retains imprinted expression because of acquired paternal DNA methylation at the sDMR and active H3K4me3 at the *Zdbf2* promoter via traversing *Liz* transcription during gastrulation.

Allele-specific DNA methylation of imprinted regions, also known as imprinted germline differentially methylated regions (gDMRs), has been studied as the best candidate for the molecular mechanism of inheriting parental-specific imprints following fertilization. Because parental imprints must be established when the parental genomes can be distinguished, investigators assayed methylation acquisition during gametogenesis, when the maternal and paternal genomes are entirely separated and can be independently epigenetically modified. Paternal-specific methylation of the gDMRs at three imprinted loci (*H19* and subsequently discovered *Dlk1-Meg3* and *Rasgrf1*) is acquired prenatally in prospermatogonia before the onset of meiosis in the male germline (Davis et al., 2000; Kato et al., 2007). In contrast, maternal-specific gDMR methylation occurs postnatally in growing oocytes, with different gDMRs (at least 21 maternal gDMRs have been identified in mice) that are methylated at a slightly different time during oocyte growth (Lucifero et al., 2004; Hiura et al., 2006). In both germlines, DNA methylation is established through the action of *de novo* DNA methyltransferase (DNMT) 3a and the accessory protein DNMT3L (Bourc’his et al., 2001; Hata et al., 2002; Kaneda et al., 2004). Although it is unclear how specific sequences are chosen for allele-specific DNA methylation in the germline, recent studies have demonstrated that histone modification across gDMR sequences provides an essential instructive step for DNMT proteins (Figures 1A,B). In oogenesis, the transcription-dependent histone marker H3K36me3 (trimethylation of histone H3 at lysine 36) guides DNA methylation over active gene bodies, leading to the establishment of all maternal methylation imprints (Kobayashi et al., 2012a; Veselovska et al., 2015; Xu et al., 2019). Transcription start sites in oocytes are often oocyte-specific (carried in part by retroviral promoters) and upstream of canonical promoters and imprinted DMRs, hence transcription-coupled DNA methylation spans these domains in an oocyte-specific manner (Chotalia et al., 2009; Brind’Amour et al., 2018). In fetal spermatogenesis, H3K36me2 (dimethylation of H3K36) shapes the gene body and intergenic DNA methylation and guides paternal methylation at the gDMRs (Shirane et al., 2020). Only in *Rasgrf1* gDMR, the Piwi-interacting RNA (piRNA) pathway and the rodent-specific DNMT3C are also responsible for the establishment of paternal DNA methylation (Watanabe et al., 2011; Barau et al., 2016). In addition to imprinted gDMRs, there are more than a thousand promoters or CpG islands on non-imprinted genes that are differentially methylated between oocytes and sperm; however, the vast majority lose their differential marks during epigenetic reprogramming events during early embryogenesis (Smallwood et al., 2011; Kobayashi et al., 2012a). After fertilization, the paternal genome is actively demethylated before the first DNA replication, whereas the maternal genome is passively demethylated throughout several rounds of DNA replication until the blastocyst stage. Imprinted gDMRs are protected from these erasure events by recruiting maintenance DNMT1 and accessory UHRF1 through the recognition of a methylated sequence motif by the zinc-finger proteins, ZFP57 and ZNF445, along with the interaction of TRIM28 with histone methyltransferases (Sharif et al., 2007; Hirasawa et al., 2008; Quenneville et al., 2011; Messerschmidt et al., 2012; Takahashi et al., 2019).

At the end of 2018, at least 260 coding and non-coding genes were found to be imprinted, and 24 imprinted gDMRs were identified in the mouse genome (Tucci et al., 2019). Many of these gDMRs act as imprinting control regions (ICRs) regulating the monoallelic expression of the neighboring solo imprinted gene and clusters of imprinted genes. The majority of maternal ICRs directly regulate a promoter for either a messenger RNA or a lncRNA by silencing one allele by DNA methylation. In contrast, paternal ICRs are not located at promoters but rather map to intergenic regions. However, the imprinting of gene clusters often involves locus-specific and complex molecular mechanisms, such as transcriptional silencing by an antisense transcript and allele-specific chromatin changes at target genes or *cis-*regulatory elements by the ICRs (Hark et al., 2000; Terranova et al., 2008; Latos et al., 2012). These imprinted genes under the control of ICRs also act as barriers to prevent mammalian embryos from parthenogenesis (Kono et al., 2004; Kawahara et al., 2007; Li et al., 2018). Thus, parent-of-origin specific DNA methylation, also called “canonical imprinting,” is considered a primary imprint marker that directly or indirectly controls most imprinted genes, which are responsible for the abnormalities of uniparental disomies or embryos.

## Non-Canonical Imprinting Is Mediated by Maternal Histone Modification

Although DNA methylation has been known to specify imprinting, the possibility that histone modifications in the gametes could also determine imprinting has also been demonstrated (Okae et al., 2014). A subset of imprinted genes is specifically paternally expressed in the placenta but not imprinted in the embryo, and the establishment of a part of such imprinted genes is independent of oocyte-specific DNA methylation, as DNMT-deficiency in growing oocytes did not affect the imprinted paternal expression of these genes in the extra-embryonic lineage (Chen et al., 2019; Hanna et al., 2019). The key gametic imprinting mark of the “non-canonical” (DNA methylation-independent) imprinting is the repressive histone mark H3K27me3 (trimethylation of H3 at lysine 27) in the oocyte, which was found to transiently imprint several loci within pre-implantation (Inoue et al., 2017a). Furthermore, H2AK119ub1 (mono-ubiquitinated histone H2A at lysine 119) was highly colocalized with H3K27me3 in oocytes, which is equalized mainly at the two-cell stage but guides maternal H3K27me3 inheritance after fertilization (Chen et al., 2021; Mei et al., 2021). Thus, H2AK119ub1 and H3K27me3, which are catalyzed by the polycomb repressive complexes (PRC1 and PRC2), mediate maternal allele-specific silencing of at least seven imprinted genes, namely *Sfmbt2*, *Phf17*, *Gab1*, *Sall1*, *Platr20* (*5133400J02Rik*), *Smoc1*, and *Slc38a4*, in mice (Figure 1C), several of which have been previously shown to play important roles in placental function and development (Itoh et al., 2000; Miri et al., 2013; Matoba et al., 2019). Maternal H3K27me3 and H2AK119ub1 are not maintained beyond pre-implantation development (Hanna et al., 2019; Chen et al., 2021; Mei et al., 2021), and transition to a more permanent epigenetic state is required to preserve paternal expression during post-implantation development (Inoue et al., 2017a; Chen et al., 2019; Hanna et al., 2019). The long terminal repeats (LTRs) of endogenous retroviral elements can act as alternative promoters for non-canonical imprinted genes and paternal allele-specific accumulation of the active histone mark H3K4me3 (trimethylation of H3 at lysine 4) occurs at these LTR promoters (Hanna et al., 2019). Finally, these LTRs are methylated on the maternal allele in extra-embryonic tissues; thus, maternally inherited H3K27me3 imprinting transitions to imprinted DNA methylation at the secondary DMRs (sDMRs) and can act as a long-term imprinting in placental linage.

Notably, oocyte-derived H3K27me3 also serves as a maternal imprint for the lncRNA Xist, triggering paternal X chromosome inactivation in mouse female pre-implantation embryos and extra-embryonic tissues (Inoue et al., 2017b). Like non-canonical imprinting at autosomal loci, X inactivation can be clonally inherited and suppress the entire chromosome through several epigenetic suppression pathways (Chen and Zhang, 2020). In addition to H3K27me3 imprinting, failure of X chromosome inactivation results in embryonic lethality, emphasizing the developmental importance of these interrelated processes. However, the functional and molecular relationship between H3K27me3-mediated non-canonical imprinting at autosomes and imprinted X chromosome inactivation or what distinguishes these strategies for biological diversity from DNA methylation-based canonical imprinting remains unresolved.

## Secondary DMRs: A Lesson From *Zdbf2* Imprinted Gene

Unlike gDMRs, imprinted sDMRs acquire allele-specific DNA methylation during embryonic development, rather than inheriting it from germ cells. Although secondary DMRs do not function as primary imprinting markers, allele-specific methylation of these regions frequently corresponds to gene silencing in a tissue-specific manner, such as *Cdkn1c* (Fan et al., 2005; Wood et al., 2010). Although sDMRs may play a role in maintaining imprinted expression (John and Lefebvre, 2011; Kobayashi et al., 2012b), they remain untested in most regions. The majority of sDMRs at canonical imprinted loci have been identified to be located within the imprinted genes or clusters and acquire allele-specific methylation by the hierarchical regulation of the gDMRs (Stoger et al., 1993; Lopes et al., 2003; Yamasaki et al., 2005; Williamson et al., 2011; Mohammad et al., 2012; Greenberg et al., 2017; Saito et al., 2018). One mechanism across several imprinted loci is the presence of a monoallelic transcript from gDMR passing through regulatory elements such as promoters and CpG islands (Ferguson-Smith, 2011). Consequently, DNMT3B targets sites of transcriptional elongation (Baubec et al., 2015), resulting in the acquisition of DNA methylation along the transcribed allele. As not all DMRs are located within transcribed regions, there must also be alternative mechanisms to establish allelic methylation at secondary loci.

Differences in the acquisition of sDMRs between embryonic and extra-embryonic lineages have been observed across several canonical imprinted domains (Lewis et al., 2004; Sato et al., 2011; Duffie et al., 2014). In particular, the DMR dynamics observed at *Zdbf2* highlight epigenetic changes in these developmental processes (Figure 1D). *Zdbf2* is a canonical, but unique, imprinted gene with paternal expression and, paradoxically, a paternal DMR near its promoter [the paradoxical finding of the paternal DMR adjacent to a paternally expressed gene was later explained through serial experiments systematically ablating epigenetic modifiers (Greenberg et al., 2017)]. Early studies of *Zdbf2* suggested that paternal DMR might be a gDMR because the DMR is methylated in the sperm and not in oocytes (Kobayashi et al., 2009). However, subsequent studies in embryos showed that paternal DNA methylation was erased in pre-implantation embryos and reset secondarily during post-implantation development (Kobayashi et al., 2012b; Duffie et al., 2014). This paternal sDMR was established by the transient monoallelic expression of a long isoform of *Zbdf2* (*Liz*, also called *GPR1AS* in humans) originating from an upstream transcription start site, which is regulated by a maternal gDMR (Kobayashi et al., 2012b, 2013; Greenberg et al., 2017). Thus, *Liz*-induced sDMR can be maintained in embryonic lineage and lead to postnatal paternal expression of *Zdbf2*. Meanwhile, *Liz* transcription is lost with the subsequent monoallelic to biallelic DNA methylation switch of the upstream maternal gDMR in embryonic tissues; conversely, the maternal gDMR remains intact throughout the post-implantation epigenetic programming in extra-embryonic tissues (Kobayashi et al., 2013; Greenberg et al., 2017). Finally, the canonical *Zdbf2* promoter and exons remain silenced because of the incomplete establishment of the paternal sDMR, and the paternal expression of *Liz* continues throughout placental development (Greenberg et al., 2017). Thus far, it remains unclear why maternal gDMR persists in extra-embryonic tissues but not in embryos.

Paternal DNA methylation at the *Zdbf2* sDMR is required to prevent the accumulation of H3K27me3, thereby conferring an active chromatin state at the adjacent *Zdbf2* promoter (Greenberg et al., 2017). It is not clearly understood what controls allele-specific DNA methylation at the sDMRs of canonical and non-canonical imprinted loci. However, further investigations into sDMRs at both canonical and non-canonical imprinted loci will provide valuable suggestions on how reprogramming or preserving factors target imprinted epigenetic marks through post-implantation development.

## Canonical and Non-Canonical Imprinting in the Other Rodents

Although mice are the primary research model used to study genomic imprinting, imprinted regions have been described in various mammals, including humans. Among the 24 gDMRsin mice, two paternal (*H19* and *Dlk1-Meg3*) and 16 maternal (*Gpr1as/Liz, Mcts2, Nnat, Nespas-Gnasxl, Gnas_exon1A, Peg10-Sgce, Mest, Nap1l5, Peg3, Snrpn, Inpp5f_v2, Kcnq1ot1, Plagl1, Grb10, Peg13*, and *Airn*) gDMRs were conserved between mice and humans (Table 1). Although some species-specific maternal gDMRs drive oocyte transcription initiation in lineage-specific LTR retrotransposons (Bogutz et al., 2019), many canonical imprinted loci are well conserved among species, and mice with deletions involving imprinted genes or ICRs are used as models for human imprinting diseases such as Prader–Willi, Angelman, Beckwith–Wiedemann, and Silver–Russell syndromes (Chang and Bartolomei, 2020). However, orthologs of non-canonical imprinted genes are not likely to be imprinted in humans. Preliminary studies in human embryos found five paternally expressed genes that may be regulated by maternal H3K27me3, but none of these have been reported to be imprinted in mice (Zhang et al., 2019). Thus, current studies to date do not provide any direct evidence for the existence of non-canonical imprinting in mammals other than mice. However, among these genes, *Sfmbt2* and *Smoc1* have been reported to show an expression biased toward one parental allele in rat and hamster placentas, respectively (Wang et al., 2011; Brekke et al., 2016). This evidence supports the hypothesis that non-canonical imprinting is conserved in rodents.

**TABLE 1:** List of identified canonically and non-canonically imprinted regions.

Type of imprinting	Mouse	Rat*^1^	Hamster*^1^	Human
Paternal gDMRs (canonical imprinting)	3 loci (*H19*, *Dlk1-Meg3*, *Rasgrf1*)	3 loci (*H19*, *Dlk1-Meg3*, *Rasgrf1*)	2 loci*^2^ (*H19*, *Dlk1-Meg3*)	2 loci (*H19*, *DLK1-MEG3*)
Maternal gDMRs (canonical imprinting)	16 common and 5 mouse (rodent)-specific loci (*Fkbp6*, *Cdh15*, *Zrsr1*, *Slc38a4*, *Impact*)	*Igf2r*, *Peg3* (common) and *Impact* (rodent-specific)	6 common loci (*Peg3*, etc.) and *Impact* (rodent-specific)	16 common and numerous human-specific loci
Non-canonical imprinting	7 loci (*Sfmbt2*, *Smoc1*, *Gab1*, etc.)	*Sfmbt2*	*Smoc1*	5 loci (*FAM101A*, etc.)

*^1^Basically, DNA methylation has not yet been well-analyzed in rats and hamsters. But imprinted regions that show imprinted expression of one or more homologous genes at each differentially methylated region (DMR) locus are listed. *^2^There is no direct evidence that Rasgrf1 is imprinted in the hamster. However, Dnmt3C, which mediates Rasgrf1 imprinting, is present in the hamster genome (Barau et al., 2016).

Although rats and hamsters are widely used for physiological, oncological, and other medical studies, mice have always been used as embryological and genetic studies models. In this situation, the number of imprinted genes identified in these rodents is limited compared to mice. However, because of the long history of laboratory animal research, numerous mouse, rat, and hamster strains have been established and maintained, and the genomes of some have been sequenced. It is possible to identify imprinting information from polymorphism information among strains (Hermsen et al., 2015).

It has already been shown that single or multiple genes are imprinted on the homologous regions of the three imprinted clusters (*H19*, *Dlk1-Meg3*, and *Rasgrf1*) that undergo paternal methylation imprinting in mice (Overall et al., 1997; Pearsall et al., 1999; Dietz et al., 2012). In addition, *Igf2r*, *Impact* [driven by a rodent-specific LTR (Bogutz et al., 2019)], and *Sfmbt2*, which are controlled by maternal imprinting in mice, are also expressed only from one parental allele in rats (Mills et al., 1998; Okamura et al., 2005; Miri et al., 2013). *Sfmbt2* is almost exclusively expressed in extra-embryonic tissues and is essential for the maintenance of trophoblast progenitors. Intriguingly, *Sfmbt2* contains a large cluster of microRNA (miRNA) genes within intron 10, and these miRNAs are also imprinted and essential for placental development (Inoue et al., 2017c). Notably, *Sfmbt2*, known to undergo non-canonical imprinting in mice, is also paternally expressed in the rat placenta in the presence of a large cluster of microRNAs (Wang et al., 2011). However, human, bovine, and pig *SFMBT2* are not imprinted and lack this block of microRNAs. These observations strengthen the argument for the recent evolution of *Sfmbt2*, in which the non-canonical imprint (and the block of miRNAs) drives its placental role in rodents.

In hamsters, reciprocal crosses between two dwarf hamsters (*Phodopus sungorus* and *Phodopus campbelli*) result in strong parent-of-origin effects on placental and embryonic growth (Brekke and Good, 2014). The expression of imprinted genes and related loss-of-imprinting has been evaluated to some extent in dwarf hamster hybrids (Brekke et al., 2016). Single-nucleotide variant-based allele-specific analysis of placental expressed genes identified 88 imprinted candidate genes in hamster autosomes. Among these, 18 genes overlapped between hamster and mice, including *Smoc1*, a non-canonical imprinted gene. Unexpectedly, *Smoc1* shows the opposite pattern of imprinting in hamster compared to mouse, with the maternal allele being expressed. This is similar to a report in human fibroblast cells, where SMOC1 showed maternal-allele specific expression (Santoni et al., 2017). While it is unclear whether this change is due to biological differences or false bias of allele-specific analysis, well-known examples of canonical paternally (*Dlk1*, *Igf2*, *Impact*, among others) and maternally (such as *H19*) expressed genes were also reidentified in hamster. *Smoc1* encodes a multi-domain secreted protein that may play a critical role in ocular and limb development (Okada et al., 2011). However, *Smoc1* is not likely associated with loss-of-imprinting in hybrid hamsters, and its functional role in placental development remains unknown.

## Conclusion

The discovery of a non-canonical imprinting mechanism mediated by histone modifications is an important finding that provides a new molecular mechanism for epigenetic transgenerational inheritance. In contrast, the diversity of canonical and non-canonical imprinting complicates our understanding of the underlying mechanisms and a better understanding of the differences among mammalian species that bridge the gap between humans and mice. For instance, the insertion of endogenous retroviral elements drives both canonical and non-canonical imprinting (Bogutz et al., 2019; Hanna et al., 2019). However, not all species-specific imprinted regions can be explained by this mechanism. Revealing the whole landscape of genomic imprinting in various rodents, such as rats and hamsters, and non-human primates would be a significant step forward in understanding the diversity of imprinting and epigenetic regulation systems.

## Author Contributions

The author confirms being the sole contributor of this work and has approved it for publication.

## Conflict of Interest

The author declares that the research was conducted in the absence of any commercial or financial relationships that could be construed as a potential conflict of interest.

## Publisher’s Note

All claims expressed in this article are solely those of the authors and do not necessarily represent those of their affiliated organizations, or those of the publisher, the editors and the reviewers. Any product that may be evaluated in this article, or claim that may be made by its manufacturer, is not guaranteed or endorsed by the publisher.

## Abbreviations

DNMT: DNA methyltransferase
gDMR: germline differentially methylated region
ICR: imprinting control region
lncRNA: long non-coding RNA
LTR: long terminal repeat
piRNA: Piwi-interacting RNA
sDMR: secondary differentially methylated region.
